# *QuickStats*: Health Center* Visit Rates,^†^ by Adults Aged ≥18 Years with Mental Health Disorder,^§^ Substance Use Disorder, or Both, by Sex — United States, 2023

**DOI:** 10.15585/mmwr.mm7401a4

**Published:** 2025-01-09

**Figure:**
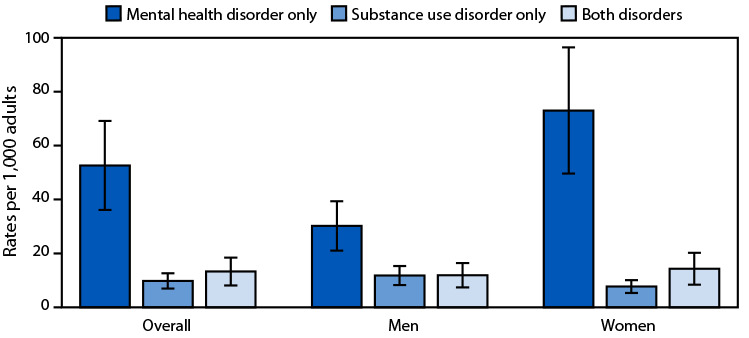
In 2023, the health center visit rate for adults with a mental health disorder was 52.6 visits per 1,000 adults. This rate was higher than the rate of visits for a substance use disorder (9.8) or both disorders (13.3). This pattern was similar for men and women. The visit rate for women with a mental health disorder (73.0) was higher than the rate for men (30.2), but the rates for visits with a substance use disorder or both disorders were similar among both men and women.

**Supplementary Table:** https://stacks.cdc.gov/view/cdc/174550

**Source:** National Center for Health Statistics, National Ambulatory Medical Care Survey Health Center Component, 2023.

For more information on this topic, CDC recommends the following link: https://www.cdc.gov/mental-health/caring/index.html

